# Commercial suture passer improves efficiency and ease of use versus conventional needle in minimally invasive thoracolumbar fascia closure: a cadaveric analysis

**DOI:** 10.1016/j.xnsj.2024.100511

**Published:** 2024-06-27

**Authors:** Michael Gallizzi, Benjamin L. Smith, Zak Kemp, Anthony N. Khoury

**Affiliations:** a Department of Spine & Neck, The Steadman Clinic, Vail, CO, United States; b Orthopedic Research Department, Arthrex, Inc., Naples, FL, United States; c Department of Spine, Arthrex, Inc., Naples, FL, United States

**Keywords:** Minimally invasive, Fascia closure, Suture-passing device, Endoscopic surgery, Spine, Thoracolumbar fascia

## Abstract

**Background:**

Low-profile suture passers have been introduced to facilitate thoracolumbar fascia closure in minimally invasive spine (MIS) surgery. The purpose of this study was to evaluate the closure time of a modern suture passer to a conventional curved need for MIS fascia closure in a cadaveric model.

**Methods:**

Six clinicians specializing in orthopedic spine surgery were recruited for the study and randomly assigned 1 cadaveric torso. Subcutaneous tissue was resected at L4-L5, replicating MIS surgery, followed by placement of a 60×18-mm or 100×18-mm tubular retractor for access. Clinicians were required to close the fascia with three unknotted, simple interrupted sutures using a swaged curved needle or suture passer (Spine Scorpion™, Arthrex, Inc., Naples, FL). The completion time was recorded, starting immediately before suturing and ending after the last pass. A time cutoff of 10 min was implemented in consideration of reasonable operating room time, and the number of achieved suture passes (of 6) were recorded. Clinicians were asked to qualitatively grade ease of use in relation to prior fascial closure experience per a 0-5 scale, where 0 is impossible and 5 is easiest.

**Results:**

The mean change in fascial closure completion time (Δ) was significantly reduced with the Spine Scorpion compared to the curved needle with the 60×18-mm retractor (Δ=5.80 min; 95% CI, 2.92-8.67 min; p=.004) and 100 × 18-mm retractor (Δ=5.28 min; 95% CI, 2.76-7.80 min; p=.003). Full closure was achieved within the time limit for all trials of the Spine Scorpion, while the standard needle achieved full closure in 67% (4 of 6) and 50% (3 of 6) of trials with the 60 × 18-mm and 100×18-mm retractors, respectively. Median ease-of-use scores with the 60×18-mm and 100×18-mm retractors, respectively, were 4.5 (range, 4-5) and 4.5 (range, 3-5) for the Spine Scorpion, and both 1.0 (range, 1-2) for the curved needle.

**Conclusion:**

Results from this laboratory investigation using a suture passer for thoracolumbar fascia closure show a significant reduction in closure time and completion of the procedure compared to a conventional curved needle.

## Introduction

The thoracolumbar fascia is a multilayer coalescence of aponeurotic and irregularly arranged collagen fiber fascial tissue that stabilizes the lumbosacral spine and envelopes the paraspinal muscles [1]. Spinal surgeries often require disruption of the thoracolumbar fascia per a variety of approaches for adequate exposure to underlying anatomy. Wound closure following spine surgery includes fascial closure with sutures to limit wound dehiscence, reduce spinal biomechanical instability, and restore hydrostatic pressure [[2], [3], [4]]. There remains a paucity of literature and no general consensus on fascia closure techniques [5,6].

Surgical treatment of spine pathologies is undergoing rapid adoption of minimally invasive surgery (MIS) techniques. Estimates project that 75% of conventional spine procedures in the United States can be accomplished using MIS techniques [7]. Key advantages include the preservation of native surrounding tissue, decreased fluid loss, earlier functional outcomes, and decreased post-operative narcotic usage [[8], [9], [10]]. Considering the reduced surgical working window, specialized tools are required to facilitate tasks. Fascial closure is accomplished using curved needles in open spine procedures; however, the procedure is complicated in the setting of tubular retractors or miniature incisions and may result in longer operating room time. Low-profile suture-passing devices have been introduced to facilitate fascial closure with 1-step suture loading and automatic passing and retrieval through the fascia.

The objective of the study was to compare the closure time of a modern suture passer to a conventional curved needle for MIS thoracolumbar fascia closure in a cadaveric model. The authors hypothesized that the improved ease of use with the suture passer would reduce fascial closure time.

## Methods

Six clinicians (5 board-certified surgeons and 1 physician assistant) specializing in orthopedic spine surgery were recruited for the study and were randomly assigned 1 cadaveric torso provided by a registered tissue bank. Institutional review board approval was not required for cadaveric research by the authors’ institution. Subcutaneous tissue was resected at L4-L5, replicating MIS surgery, followed by placement of a 60 × 18-mm or 100 × 18-mm tubular retractor (Arthrex, Inc.) for access. The incision size was slightly less than the tubular retractor so it can be naturally supported by the peripheral tissue, along with an external retractor holder. Fascia was maintained and separated with a simple vertical incision. The retractor did not have to be replaced between attempts. Devices were alternated between the evaluators and no visible perforations or substantial damage was observed for all evaluators between trials.

**Fig. 1:**
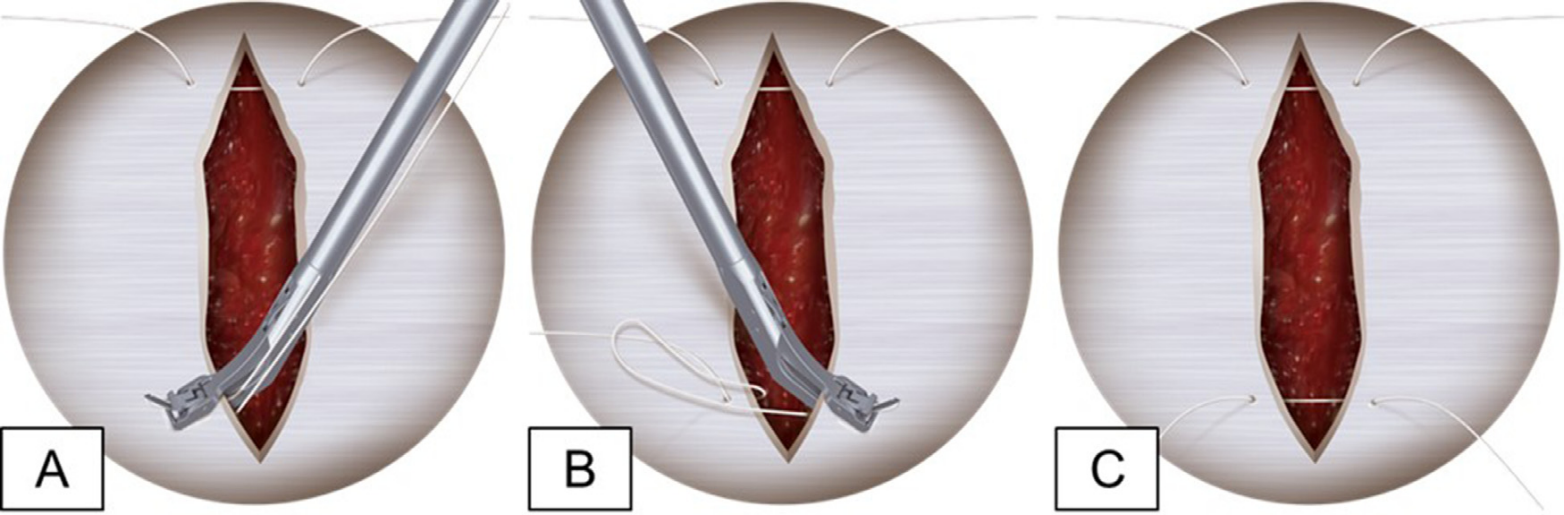
Process of passing fascial closure stitches with the Spine Scorpion™ suture passer (A-C).

Clinicians were required to close the fascia with 3 unknotted, simple interrupted sutures (2-0 FiberWire®; Arthrex, Inc.) using a swaged curved needle or suture passer (Spine Scorpion™; Arthrex, Inc.; Naples, FL; USA) (Fig. 1) with both retractor sizes. The clinician was first trained by a product specialist until they were comfortable using the closure instrument on a synthetic fascia model. Training consisted of an explanation of device operation and usage, per the manufacturer's instruction, followed by a live demonstration. A surgical technique animation is available on the manufacturer's website [11]. Additionally, a synthetic fascia model was made available for training until the clinician felt fully comfortable with its use. The environment replicated an operating room, with instrumentation, a surgical microscope (Haag-Streit; Koeniz, Switzerland), and an assistant provided. Devices were tested in an alternating manner, with sutures removed between trials.

The completion time was recorded, starting immediately before suturing and ending after the last pass. A time cutoff of 10 min was implemented in consideration of reasonable operating room time, and the number of achieved suture passes (of 6) were recorded. Clinicians were asked to qualitatively grade ease of use in relation to prior fascial closure experience per a 0-5 scale, where 0 is impossible and 5 is easiest.

### Statistical analysis

Paired t-tests (α=0.05) were performed in SigmaPlot (Systat Software; San Jose, CA, USA) for completion time comparisons. Shapiro-Wilk normality tests confirmed the paired data was normally distributed. The sample size of 6 was selected via *a priori* power analysis to reach a power of 0.80 using comparative device pilot data.

## Results

**Fig. 2:**
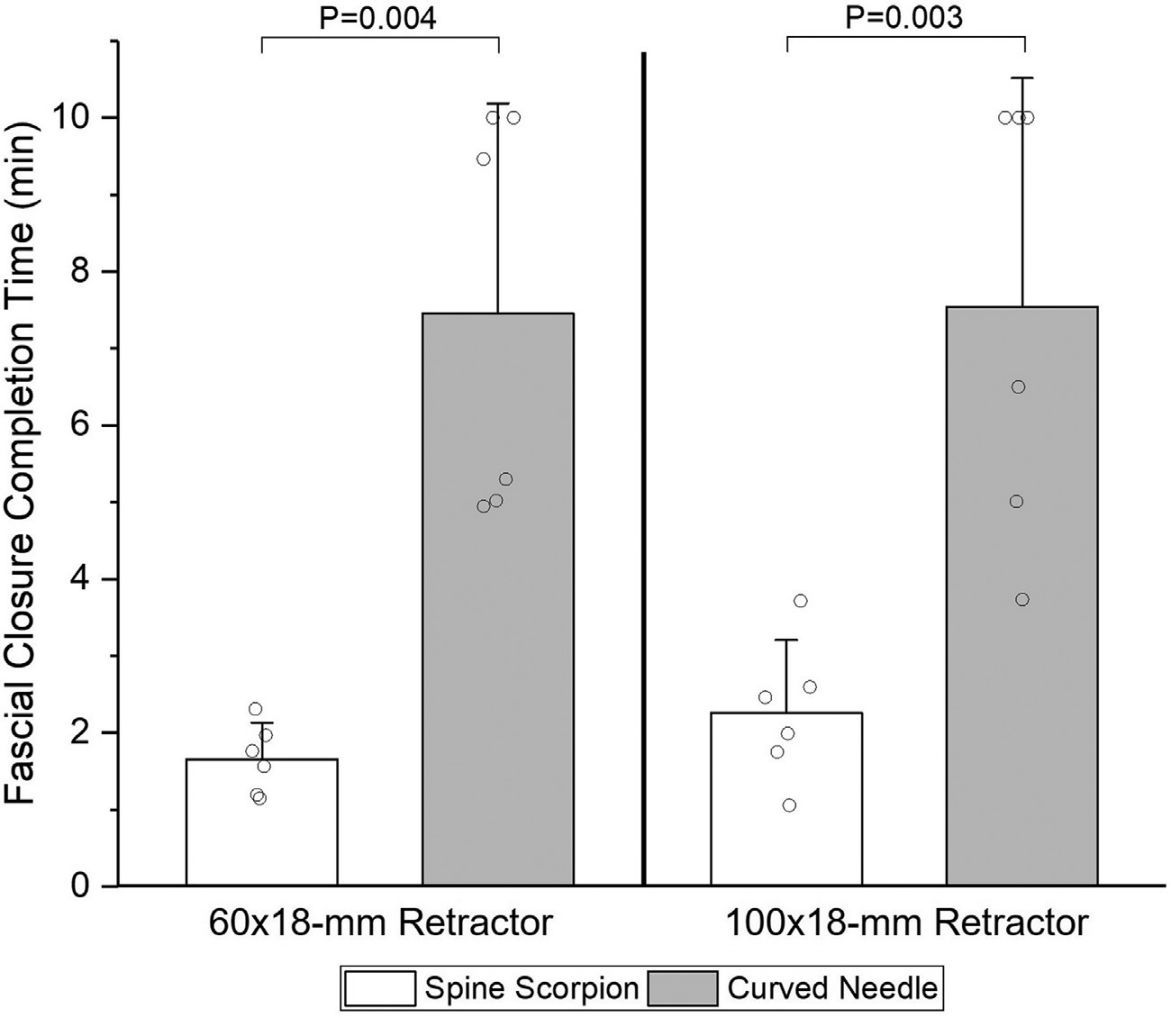
Fascial closure completion time by six clinicians using either a Spine Scorpion™ suture passer or a standard curved needle, with a 10-min cutoff. Mean with 95% CI error bar.

The mean change in fascial closure completion time (Δ) was evaluated for all participants. The Spine Scorpion significantly reduced the fascial closure completion time versus the curved needle with the 60 × 18-mm retractor (Δ=5.80 min; 95% CI, 2.92-8.67 min; p=.004) and 100 × 18-mm retractor (Δ=5.28 min; 95% CI, 2.76-7.80 min; p=.003) (Fig. 2). Full closure was achieved within the time limit for all trials of the Spine Scorpion, while the standard needle achieved full closure in 67% (4 of 6) and 50% (3 of 6) of trials with the 60 × 18-mm and 100 × 18-mm retractors, respectively. Median ease-of-use scores with the 60 × 18-mm and 100 × 18-mm retractors, respectively, were 4.5 (range, 4-5) and 4.5 (range, 3-5) for the Spine Scorpion, and both 1.0 (range, 1-2) for the curved needle.

**Fig. 3:**
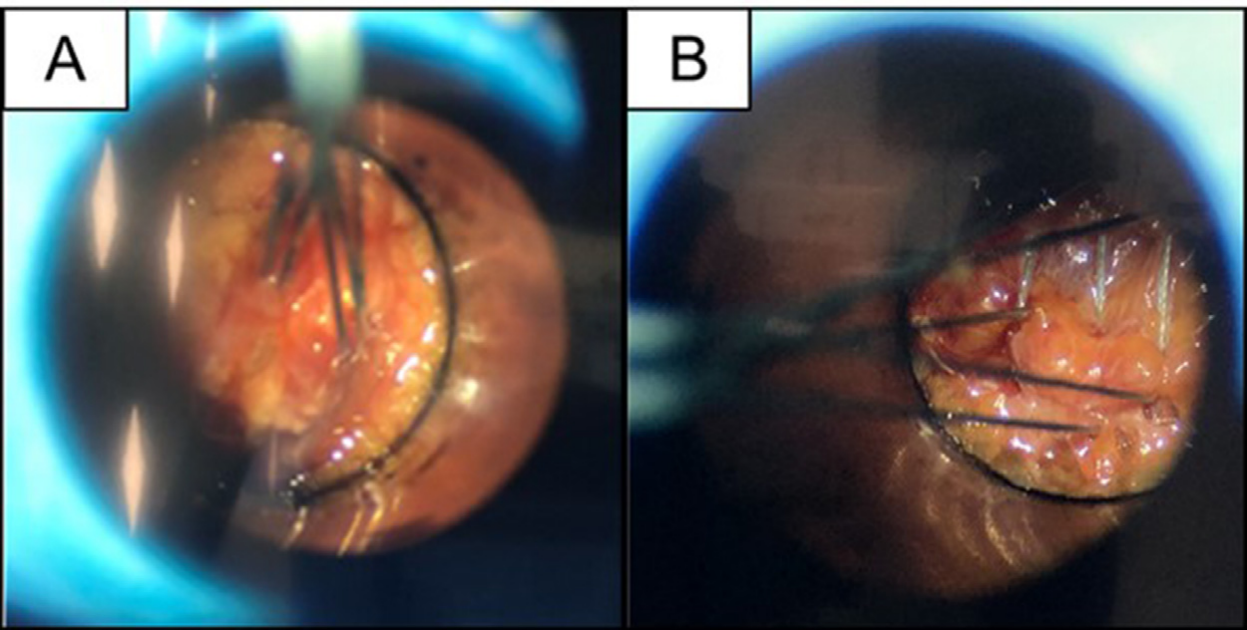
Representative photo showing (A) centralized sutures from the curved needle and (B) broadly spaced sutures achieved with the Spine Scorpion™ suture passer.

Participants noted broader suture placement with the Spine Scorpion, as it could maneuver the full tube area while the curved needle could not (Fig. 3).

## Discussion

The principal finding was that the Spine Scorpion suture passer reduced closure time by up to 75% and improved ease of use for MIS thoracolumbar fascia closure versus curved needles. MIS fascial closure with standard curved needles presents challenges in constrained working areas [6], evidenced by 5 unsuccessful attempts at completion within the time allotment. Implementation of an easily maneuverable suture passer within an MIS workflow will alleviate surgeon stressors, improve operating room efficiencies, and possibly minimize patient complications by securely closing fascia.

Access to the lumbar spine with tubular retractors was first described by Foley et al. in 1999 [12] and signaled an important shift in MIS surgery [9]. Compared to a midline incision, tubular retractors minimize disruption of muscle and nervous structures, which may translate to reduced perioperative pain, hospitalization, and blood loss [9,13]. The narrow working channels of the retractors require dedicated devices to accomplish tasks. In the presented study, with a curved needle, only 67% of participants were successful in closing the fascia through a 60 × 18 mm retractor and 50% through a 100 × 18 mm retractor within the 10-minute time frame provided. Comparatively, all participants successfully completed the fascial closure with the suture passer and communicated the advantage of broader suture placement over the closure window. These advantages not only improve postoperative patient outcomes [9], but also may alleviate surgeon stressors.

The demonstrated reduction in operating room time with the suture passer is important when considering an expanding field with a sharp learning curve of new devices and techniques. The cost of operating room time is currently estimated to range from $14.50 to $131.65 per minute [14]. Results from this study collectively suggest the suture passer could provide better outcomes per healthcare dollar, which is the ultimate goal of value based practice.

This study is not without limitations. The clinicians were provided training at the time of evaluation; therefore, the results do not consider a learning curve that may be associated with endoscopic suture passing over a time duration in the clinical setting. Further, the use of a synthetic fascial training model and cadaver may not fully replicate the true surgical experience, warranting additional clinical work.

## Conclusion

Results from this laboratory investigation using a suture passer for thoracolumbar fascia closure show a significant reduction in closure time and completion of the procedure compared to a conventional curved needle.

## Summary sentence

Thoracolumbar spine fascia closure with a suture passer improves efficiency and ease of use compared to curved needle.

## Declaration of competing interest

One or more authors declare potential competing financial interests or personal relations as specified on required ICMJE-NASSJ Disclosure Forms.

## Appendix Supplementary materials

Image, application 1
