# Supplementary material for: Commercial suture passer improves efficiency and ease of use versus conventional needle in minimally invasive thoracolumbar fascia closure: a cadaveric analysis
Source: N Am Spine Soc J. 2024 Jun 27;19:100511. doi: 10.1016/j.xnsj.2024.100511 (PMC11326955; doi:10.1016/j.xnsj.2024.100511)
Supplement: Supplementary file 1 [file mmc1.docx]

—-—-—-—

Reply above this line.

Eliana Borda commented:

Hello Anthony,

Thanks for contacting us. Your request has been approved to use Figure 14 in the Spine Scorpion suture passer technique guide ([Spinal Fascia Closure Using the Spine Scorpion Suture Passer (arthrex.com)](https://www.arthrex.com/resources/LT1-000220-en-US/spinal-fascia-closure-using-the-spine-scorpion-suture-passer?referringteam=spine)) to support the in-house research study.

Please let us know if you have any questions.

Kind regards,

Eliana Borda

Arthrex, Inc.’s hours of operation are Monday through Friday, 8:00 am to 5:00 pm (EST).

Marketing materials such as medical illustrations, photos, brochures, videos, animations, and techniques are the property of Arthrex. Marketing materials may only be used for the specific use approved by Arthrex. Express permission to utilize Arthrex marketing materials must be provided by Arthrex and is only provided on a per request basis. Healthcare Professionals wishing to utilize Arthrex assets must request Arthrex assets via AskMarketing through the [Marketing Materials Request Form](https://www.arthrex.com/corporate/contact-us/marketing-material-request). Altering Arthrex assets in any way is strictly prohibited.


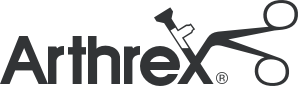


**AskMarketing Team**
1 Arthrex Way
Naples, FL 34108
[www.Arthrex.com](https://urldefense.com/v3/__https:/arthrex.atlassian.net/www.arthrex.com__;!!P192cPdC!hbhvF-m9Q47g-iZNowA9FLDYQr5MZ8Eg8haP5h94g8-cH17oibZ4e2cmHoqWznk2NQUm0V_RT_1pEzV90d_5gTGHuMo$)

Toll-Free       800-933-7001

[View request](https://urldefense.com/v3/__https:/arthrex.atlassian.net/servicedesk/customer/portal/70/MKT-4112?token=eyJ0eXAiOiJKV1QiLCJhbGciOiJIUzI1NiJ9.eyJ0Z3QiOiJhbm9ueW1vdXMtbGluayIsInFzaCI6IjBjMTI0MmE3OGQ1N2VlNTNmNjVlOTMxNTBjNmQyNDUxNzJhZGRmMzVmNGU3Y2QxYWUxNDhjY2ZlNjk0OWJkMTkiLCJpc3MiOiJzZXJ2aWNlZGVzay1qd3QtdG9rZW4taXNzdWVyIiwiY29udGV4dCI6eyJ1c2VyIjoiMjMyMTQiLCJpc3N1ZSI6Ik1LVC00MTEyIn0sImV4cCI6MTcwMTQ2Mzc4MCwiaWF0IjoxNjk5MDQ0NTgwfQ.DC0FArVCgemw2R0YwOb_WKUnOzTkm1RPZTp982qxy2Q&sda_source=notification-email__;!!P192cPdC!hbhvF-m9Q47g-iZNowA9FLDYQr5MZ8Eg8haP5h94g8-cH17oibZ4e2cmHoqWznk2NQUm0V_RT_1pEzV90d_5DRTChic$) · [Turn off this request's notifications](https://urldefense.com/v3/__https:/arthrex.atlassian.net/servicedesk/customer/portal/70/MKT-4112/unsubscribe?jwt=eyJ0eXAiOiJKV1QiLCJhbGciOiJIUzI1NiJ9.eyJ0Z3QiOiJhbm9ueW1vdXMtbGluayIsInFzaCI6IjhlYmJkM2JhOGIxZjlhMWI1NzQ2ZGFlYmIwY2I2NzkyOThmZGU3Y2FhNGJkMDAyNjAyOTI3MTUyMGUwOWYwYTIiLCJpc3MiOiJzZXJ2aWNlZGVzay1qd3QtdG9rZW4taXNzdWVyIiwiY29udGV4dCI6eyJ1c2VyIjoidWc6MjAxOGUwZjMtMDlkYi00ZmIwLWJmZWEtMDA3YzY0NmFhYzE4IiwiaXNzdWUiOiJNS1QtNDExMiJ9LCJleHAiOjE3MDE0NjM3ODAsImlhdCI6MTY5OTA0NDU4MH0.orCNw6rkw6OOtH8dU6Cd7CQaiZk0mJvlV9sf7FDCdF0__;!!P192cPdC!hbhvF-m9Q47g-iZNowA9FLDYQr5MZ8Eg8haP5h94g8-cH17oibZ4e2cmHoqWznk2NQUm0V_RT_1pEzV90d_5oPIEl1M$)

This is shared with Anthony Khoury, Benjamin Smith, and Ask Marketing.

[Powered by Jira Service Management](https://urldefense.com/v3/__https:/www.atlassian.com/software/jira/service-desk/powered-by?utm_medium=jira-in-product&utm_source=jira_service_desk_email_footer&utm_content=arthrex__;!!P192cPdC!hbhvF-m9Q47g-iZNowA9FLDYQr5MZ8Eg8haP5h94g8-cH17oibZ4e2cmHoqWznk2NQUm0V_RT_1pEzV90d_5O3DDNR0$)

Sent on November 3, 2023 4:49:40 PM EDT
